# Importance of soil amendments with biochar and/or *Arbuscular Mycorrhizal* fungi to mitigate aluminum toxicity in tamarind (*Tamarindus indica* L.) on an acidic soil: A greenhouse study

**DOI:** 10.1016/j.heliyon.2022.e09009

**Published:** 2022-02-23

**Authors:** Ndiaye Ibra Ndiate, Cai Li Qun, Jackson Nkoh Nkoh

**Affiliations:** a College of Resources and Environmental Sciences, Gansu Agricultural University, Lanzhou, 730070, China; b Gansu Provincial Key Laboratory of Arid Land Crop Science, Gansu Agricultural University, Lanzhou, 730070, China; c Organization of African Academic Doctors, Off Kamiti Road P.O. Box 25305-00100, Nairobi, Kenya; d State Key Laboratory of Soil and Sustainable Agriculture, Institute of Soil Science, Chinese Academy of Sciences, P.O. Box 821, Nanjing, 210008, China

**Keywords:** *Tamarindus indica*, Biochar, *Rhizophagus fasciculatus*, *Rhizophagus aggregatus*, *Rhizophagus irregularis*, Mycorrhization, Soil acidity

## Abstract

*Tamarindus indica* L. is a forest plant species widely used in semi-arid regions and has an important socio-economic role. A 90 d greenhouse pot experiment was conducted to evaluate the efficiency of soil amendments with biochar and/or three *Arbuscular Mycorrhizal* Fungi (AMF) strains; *Rhizophagus fasciculatus* (Rf), *Rhizophagus aggregatus* (Ra), *and Rhizophagus irregularis* (Ri) on *T. indica* grown under aluminum stress. The amendments consisted of 5% biochar and 20 g kg^−1^ AMF as (i) control; (ii) biochar; (iii) biochar + Rf; (iv) biochar + Ra; (v) biochar + Ri; (vi) Rf; (vii) Ra; (viii) Ri. The treatments with biochar significantly (*P* < 0.05) increased soil pH and reduced the content of soil exchangeable Al^3+^ relative to the control and exclusive AMF treatments. All the treatments improved total nitrogen and phosphorus uptake by roots and shoot of *T. indica* and resulted in improved plant growth and root/shoot dry weight. The ability of biochar to enhance the soil's water-holding capacity played a key role in improving the intensity of mycorrhization. Overall, biochar amendments significantly improved the photosynthetic potential of *T. indica* and the activities of antioxidant enzymes compared to other treatments. Thus, the combined effects of enhanced (a) soil physicochemical parameters, (b) mycorrhization, (c) nutrient uptake, (d) photosynthetic potential, and (e) antioxidant activities played an important role in mitigating Al-related stress to improve the growth of *T. indica*. Therefore, the application of biochar in combination with AMFs can serve as a strategy for ensuring plant biodiversity in acid and Al-toxic soils in arid and semi-arid regions in Africa.

## 1 Introduction

World biodiversity is currently subject to drastic changes that have resulted in a reduction of the terrestrial biological wealth; of which, developing countries are on the negative end [1, 2]. Among the components of the biosphere, plants are particularly sensitive to these changes [3, 4]. Forest trees play an important role in ensuring the well-being of the populations; particularly those living in rural areas of semi-arid regions [5]. In most parts of West Africa, the fruits and leaves of some forest tree species provide trace elements, vitamins, and proteins. These nutrients are rare in cereals but essential to maintain a dietary balance and are also a source of income and traditional medicines [6, 7, 8]. However, abiotic stresses have caused a significant dysfunction in the bio-functioning of the ecosystems and in the long term, would no longer allow plant cover to ensure its sustainability and development [9].

*T. indica* is a monotypic genus in the subfamily *Caesalpinioideae* of the *Leguminosae* (Fabaceae), and it is an important multipurpose fruit tree species that can adapt to many environments. *T. indica* is moderately big, up to 24 m tall and 7 m in girth and the fruit pulp has high levels of fats and oils, fiber, and many other components like protein, ash, vitamins (particularly vitamin C), and minerals [10, 11, 12]. Due to the high variety of phytochemicals, *T. indica* has been shown to have antibacterial, antidiabetic, antioxidant, antiasthmatic, antimalarial, sedative, anti-hyperlipidemic, and hepatoprotective properties [13, 14]. Based on these beneficial properties, *T. indica* has undeniably huge potential [15].

The percentage of acidic soils in the tropics is fast increasing due to an increase in anthropogenic activities and excessive use of N-based fertilizers which results in soil acidification [16]. Soil infertility associated with soil acidification is mostly caused by toxic aluminum (Al) which has been observed to harm plant biodiversity [17]. Mossor-Pietraszewska [18] and Shi et al. [19] observed that high concentrations of Al in soils negatively affect plants by inhibiting root elongation, and under such conditions, important nutrients (e.g. Mg, Ca, K, P, N) become deficient [20]. Soil pH is the most important factor influencing the form of Al in soils, thus, it determines how phytotoxic and damaging it is to Al-sensitive plant roots [21]. The content of Al in soil solution is controlled by dissolution reactions of Al-bearing minerals, which is also largely influenced by soil pH [22].

The soil microflora has a consistent role in the spatial and temporal organization of the ecosystem evolution [23]. It plays an important role in ensuring the co-existence of plants and the dynamics of the ecosystem and its productivity [24]. Within the microbial communities populating the soil, there are symbiotic microorganisms including AMF which has a close relationship with plants [25, 26, 27, 28, 29, 30]. AMF is a key component in the biological mechanisms ensuring the resilience capacity of an ecosystem and can be used in strategies for stress mitigation [31]. AMF performs well in associations with forest plants and protects them from abiotic stress by improving their survival in the early development stages [32, 33, 34]. The symbiotic association in plant roots with AMF is one strategy for improving the host plant's tolerance to metal stress [35], and this significantly mitigates metal-related stress by enabling plant nutrient availability and affecting the fate of metals in plants and soil [36]. Also, the extraradical mycelium of AMF plays an important role as a plant root extension and can reach beyond the root depletion zone to better explore the soil for better water and nutrient uptake [37]. Therefore, the choice of an appropriate combination of plant-AMF species may be a potential strategy for the phytostabilization of high metal concentration in soils [38, 39]. Thus, this strategy can be very important in improving forest plants response to acidification and Al-related stresses in tropical soils in Africa and for improving plants biodiversity.

The use of nutrient-rich biochar in mitigating the negative effects of abiotic stress on forest species have been studied [40, 41, 42]. According to previous reports, plants under biochar amendment have developed advanced mechanisms to minimize stress damage or re-establish growth by modifying the plant metabolism [43]. Biochar treatment is known to alleviate Al toxicity by reducing soil acidity and enhancing soil fertility [44]. Amending soils with biochar has gained a lot of attention in the last two decades due to its role in improving (i) carbon (C) sequestration and mitigating global warming, (ii) soil moisture-holding capacity, (iii) soil nutrients such as Mg, Ca, K, P, N, and (iv) immobilization of pollutants in soils [45, 46, 47, 48]. Therefore, the application of biochar in forest acid soils in Africa might be a sustainable method to enhance soil fertility and improve biodiversity. From existing knowledge, little research has been done to evaluate the effect of biochar and/or AMF in improving the growth of forest plants in Africa. Thus, this study was designed to determine the individual performance of biochar or in combination with AMF in promoting the growth of *T. indica*. Specifically, we studied the effects of the treatments on *T. indica* growth under Al stress in an acidic soil by considering (a) the plant height, shoot and root dry weight; (b) the intensity of mycorrhization; (c) plant nutrient contents, (d) photosynthetic pigments and antioxidant enzymes activity, and (e) the changes in soil physicochemical properties.

## 2 Materials & methods

### 2.1 Experimental site, soil, AMF, biochar, and seeds

This study was carried out in Dakar, Senegal, West Africa. The climate in this area is semi-arid and hot, with a brief wet season and a long dry season. In Dakar, the rainy season starts from July to October and the remaining eight months are known as the dry season; an annual rainfall of about 391.6 mm. The moderately hydromorphic gley soil used in this experiment was collected from the 20–40 cm depth from an agricultural area in Sangalkam, Rufisque, Senegal. Soil material was sampled, air-dried in the site, transported to the laboratory and sieved with a 250 μm mesh for chemical analysis. The soil physicochemical properties were determined as described below (subsection 2.1) and are given in Table 1.

**Table 1:** Basic characteristics of the soil and biochar.

Properties	Soil	Biochar
Sand (%)	88.8	-
Silt (%)	5.8	-
Clay (%)	5.4	-
OM (%)	1.20 ± 0.03	-
TN (g/kg)	1.09 ± 0.03	45.82 ± 0.63
NH_4_–N (mg/kg)	46.63 ± 2.38	33.18 ± 0.61
NO_3_–N (mg/kg)	23.25 ± 0.20	96.84 ± 0.34
TOC (%)	0.64 ± 0.42	11.54 ± 0.11
Ex. K (g/kg)	0.98 ± 0.04	5.15 ± 0.15
P-OLSEN (g/kg)	26.88 ± 0.97	68.69 ± 0.29
Ex. Ca (g/kg)	32.12 ± 0.19	31.56 ± 0.08
Ex. Mg (g/kg)	2.20 ± 0.28	13.44 ± 0.12
Ex. Al (mg/kg)	25.85 ± 0.42	-
pH	5.50 ± 0.14	7.62 ± 0.07

OM: organic matter, TN: total nitrogen, TC: total carbon, TOC: total organic carbon, Ex. = exchangeable.

Three AMF species; *Rhizophagus fasciculatus* (Rf), *Rhizophagus aggregatus* (Ra), *and Rhizophagus irregularis* (Ri) were obtained from the Common Mycorrhiza Laboratory, Research Institute for Development (Dakar, Senegal). They were multiplied using *Zea mays* (L) in sterilized soil in a greenhouse for four months. On average, the colonization of Rf, Ra, and Ri was 95.3, 93.2, and 94.3%, respectively. The AMF inoculums consisted of colonized roots fragments, soil containing spores and extraradical hyphae as previously described by Yang et al. [49].

Corn straw-derived biochar was obtained from an agricultural shop in Dakar. The chemical compositions of the biochar as provided by the shop (*Niayes* Sarraut) are given in Table 1. Also, the seeds of *T. indica* were provided by the National Agency of Senegal Great Green Wall, Dakar, Senegal. The seeds of uniform size were kept at 4 ᵒC for 24 h, and later in running water for 30 min according to the local practices.

### 2.2 Experimental treatments

A pot experiment was conducted for 90 days (from July to October 2019) in the greenhouse of the College of Technical Sciences, Dakar University (Fann Town, Dakar City). The greenhouse was an open-side greenhouse that was covered on the top with a polythene cover to prevent rainwater from irrigating the plants. For each treated pot containing 4.0 kg sterilized soil, an amount of 20.0 g AMF inoculum was mixed with the soil with/without 5% biochar and three imbibed seeds of *T. indica* were sown. The experiment was carried out in a completely randomized design with the following treatments: (1) biochar alone (B1 + R0)*;* (2) biochar and *Rhizophagus fasciculatus* (B1 + Rf); (3) biochar and *Rhizophagus aggregatus* (B1 + Ra); (4) biochar and *Rhizophagus irregularis* (B1 + Ri); (5) *Rhizophagus fasciculatus* alone (Rf + B0); (6) *Rhizophagus aggregatus* alone (Ra + B0); (7) *Rhizophagus irregularis* alone (Ri + B0)*;* and (8) control (B0 + R0). Each treatment was repeated ten times making a total of 80 round plastic pots (21 cm in diameter ×16 cm in height). A gauze was used to cover the holes at the bottom of the pots to prevent soil loss. The irrigation regime was done as per the requirement to maintain adequate moisture necessary for seedling growth.

### 2.3 Data collection and analysis

#### 2.3.1 Soil characterization

Soil pH was determined using a METTLER TOLEDO Desktop pH meter after the soil sample was equilibrated in distilled water (1:5). The percentages of sand, silt and clay in the soil sample were determined by the Bouyoucos-hydrometer method [50]. The content of organic matter (OM) and total organic carbon (TOC) were estimated by the Walkley–Black method and by the Wet Oxidation method [51], respectively. Total nitrogen (TN) and phosphorus (TP) were determined by the Kjeldahl method [52] and Olsen method [53], respectively. Also, the content of NH_4_–N and NO_3_–N were quantified by the FIAstar™ 5000 Analyzer after extracting the soil with 2.0 M KCl. The exchangeable K, Mg and Ca were evaluated by the Ammonium Acetate extraction method while exchangeable Al was extracted using 1.0 M KCl and determined by ICP-AES [54].

#### 2.3.2 Plant growth parameters

The height of 5 plants from each treatment was recorded by holding the pole close to the stem of the plant. Plant height was determined from the ground level to the leaves base of the highest and fully expanded leaf. The ten plants from each treatment were carefully removed from the soil and washed with distilled water before being manually separated into the root and shoot parts. The plant shoots and roots were dried in an oven at 65 °C for 72 h, and the dry weight (DW) was recorded thereafter.

#### 2.3.3 Plant nutrient uptake, photosynthetic pigments, and the intensity of mycorrhization (I%)

Plant N and P contents were estimated following Abeer et al. [55]. The photosynthetic pigments contents of leaves were also determined after the experiment by estimating the (i) chlorophylls content per the method of Arnon [56] (Eqs. (1) and (2)) and carotenoids content by Khan et al. [57] (Eq. (3))

1 Chlorophyll a (mg g^−1^ FW) = (0.0127 ∗ A_663_) – (0.00269 ∗ A_645_)

2 Chlorophyll b (mg g^−1^ FW) = (0.0229 ∗ A_645_) – (0.00468 ∗ A_663_)

3 Carotenoids (mg g^−1^ FW) = (1000 ∗ A_470_ – 2.270 ∗ Chl. a – 81.4 ∗ Chl. b)

Where A_663_, A_645_, A_470_ are absorbance at 663, 645, and 470 nm, respectively while Chl. a and Chl. b are contents of chlorophyll a and chlorophyll b from Eqs. (1) and (2), respectively.

Root staining was performed according to the method of Philips and Hayman [58] to assess mycorrhization rates. The fresh roots of *T. indica* were thoroughly rinsed with tap water to remove soil particles. They were then placed in test tubes in a 10% w/v KOH solution for discoloring the roots and to empty the cytoplasmic contents. The tubes containing the roots and KOH were heated in a water bath at 90 °C for 1 h. After heating, the roots were then rinsed to remove KOH and placed in a 0.05% w/v Trypan blue solution. The tubes containing the roots soaked in Trypan blue were placed again in a water bath at 90 °C and heated for 30 min. For each sample, 20 root fragments of approximately 1 cm were mounted between slides and coverslip; four slides were made for each sample. The roots were crushed in glycerol and observed under the microscope. The presence of hyphae, vesicles or arbuscles in the root confirms mycorrhizal colonization of the root sample. The estimation of root colonization by arbuscular mycorrhizal fungi was done using the method of Trouvelot et al. [59], while the intensity of mycorrhization (I%) was estimated according to Eq. (4).

4 I% = (95n^5^ + 70n^4^ + 30n^3^ + 5n^2^ + n^1^) /total number of fragments observed

where;

n^5^ = number of fragments noted 5
n^4^ = number of fragments noted 4
n^3^ = number of fragments noted 3
n^2^ = number of fragments noted 2
n^1^ = number of fragments noted 1

#### 2.3.4 Antioxidant enzymes activity

Plant fresh leaves were collected during harvesting for protein extracts. The samples (1 g, ground leaves samples) were frozen in liquid nitrogen, lyophilized, and homogenized in 2 mL of 0.1 mM potassium phosphate (pH 7.8). The suspension was centrifuged for 15 min at 4 °C at 12, 000 g. The supernatants were used for the assay of enzymatic activity. Catalase activity in *T. indica* leave extract was determined by the method of Aebi [60]. The disappearance of hydrogen peroxide (H_2_O_2_) was measured by determining the absorbance decrease at 240 nm for 2 min. The activity of catalase was calculated by using an extinction coefficient of 40 M^−1^ cm^−1^. Catalase (CAT) activity was expressed as nmol min^−1^ g^−1^ fresh weight. Guaiacol peroxidase (POD) activity was obtained according to the formula: FW (mM/min/g) = changes in absorbance/min. ∗ total volume (mL)/Extinction coefficient ∗ volume of samples (mL) [61]. Ascorbate peroxidase (APX) activity was calculated according to the formula- FW (mM/min/g) = changes in absorbance/min. ∗ total volume (mL)/Extinction coefficient ∗ volume of samples (mL). The APX was calculated using an extinction coefficient of 2.8 mM^−1^ cm^−1^ [62].

### 2.4 Statistical analysis

Data were analyzed by two-way analysis of variance (ANOVA) using Genstat statistical software (ver.12). Significant differences among treatments were calculated by Duncan's multiple range tests (*P* < 0.05).

## 3 Results

### 3.1 Basic properties of the soil before and after amendments and growth of *T. indica*

Table 1 shows that the hydromorphic gley soil used in this study contained predominantly sand (88.8%) with little clay (5.4%) and silt (5.8%). The soil is acidic with pH_water_ 5.50, the sum of exchangeable base cations (Ca + Mg + K) of 35.3 g kg^−1^, and soluble Al content of 25.85 mg kg^−1^. After amending soils with AMF or biochar and growing *T. indica*, the soil physicochemical properties were affected (Table 2). All treatments with biochar showed a significant (*P* < 0.05) positive effect on total organic carbon (TOC) and total nitrogen (TN) while treatments with AMF alone showed a positive effect only for TOC but negatively affected TN relative to the control (Table 2). The contents of NH_4_–N and NO_3_–N were significantly increased by the biochar treatments but not for treatments containing AMF alone compared to the control. Specifically, the contents of NH_4_–N and NO_3_–N in the control treatment after harvest were 42.63 and 13.26 mg kg^−1^ as opposed to 52.67 and 34.04 mg kg^−1^ for biochar treatment and 53.05 and 34.89 mg kg^−1^ for B1 + Rf treatment, respectively (Table 2). Comparatively, the Rf alone treatment has corresponding values of 42.88 and 14.18 mg kg^−1^, respectively. Thus, all treatments with biochar significantly (*P* < 0.05) increased the contents of NH_4_–N and NO_3_–N relative to the control while AMF treatments did not. The content of Olsen P was not significantly affected by any of the treatments while the sum of exchangeable base cations (Mg + Ca + K) increased by 10.53, 9.95, 9.95, and 8.71% for biochar, B1 + Rf, B1 + Ra, and B1 + Ri, respectively. For the treatments with AMF alone, the contents of exchangeable base cations increased by 2.31, 2.82, and 3.0% for Rf + B0, Ra + B0, and Ri + B0, respectively. Also, Table S1 shows that biochar and its interaction with AMF had a significant positive relationship with TN, NH_4_–N, and NO_3_–N while AMF did not.

**Table 2:** Effect of soil amendments on soil chemical characteristics after harvesting of *T. indica*.

*Mycorrhization*	*Biochar amendment*	TN (g kg^−1^)	NH_4_–N (mg kg^−1^)	NO_3_–N (mg kg^−1^)	TOC (%)
*R0*	*B0*	0.79 ± 0.13b	42.63 ± 1.86b	13.26 ± 0.413c	0.79 ± 0.06c
	*B1*	1.69 ± 0.15a	52.67 ± 0.29a	34.04 ± 0.036b	1.203 ± 0.01ab
*Rf*	*B0*	0.63 ± 0.08b	42.88 ± 0.8b	14.18 ± 0.758c	0.95 ± 0.05abc
	*B1*	1.66 ± 0.125a	53.05 ± 0.96a	34.89 ± 1.652ab	1.18 ± 0.10ab
*Ra*	*B0*	0.55 ± 0.20b	42.32 ± 0.59b	13.72 ± 0.241c	0.91 ± 0.04bc
	*B1*	1.66 ± 0.23a	53.33 ± 1.53a	34.73 ± 1.207ab	1.19 ± 0.27ab
*Ri*	*B0*	0.61 ± 0.18b	42.96 ± 0.36b	14.26 ± 0.341c	1.06 ± 0.16abc
	*B1*	1.67 ± 0.058a	53.33 ± 1.72a	35.89 ± 1.066a	1.28 ± 0.37a
		P-OLSEN (g kg^−1^)	Ex. Ca (g kg^−1^)	Ex. Mg (g kg^−1^)	Ex. K (g kg^−1^)
*R0*	*B0*	22.98 ± 0.75a	30.17 ± 0.26a	1.92 ± 0.13b	0.87 ± 0.02a
	*B1*	23.16 ± 1.27a	32.08 ± 0.11a	3.48 ± 0.08a	0.87 ± 0.04a
*Rf*	*B0*	21.71 ± 0.34a	30.78 ± 0.51a	1.97 ± 0.70b	0.97 ± 0.11a
	*B1*	23.23 ± 1.22a	31.77 ± 1.24a	3.58 ± 0.42a	0.89 ± 0.11a
*Ra*	*B0*	21.57 ± 0.90a	30.79 ± 1.10a	2.08 ± 0.14b	1.02 ± 0.23a
	*B1*	24.07 ± 0.75a	31.53 ± 0.77a	3.75 ± 0.49a	0.96 ± 0.06a
*Ri*	*B0*	22.44 ± 1.5a	30.90 ± 0.48a	2.0 ± 1.0b	1.05 ± 0.17a
	*B1*	23.36 ± 1.36a	31.0 ± 0.80a	3.78 ± 0.13a	1.05 ± 0.06a

B0 (absence of biochar amendment), B1 (soil treated with 5% biochar), R0 (absence of Mycorrhization), Rf (soil treated with 20 g *Rhizophagus fasciculatus*), Ra (soil treated with 20 g *Rhizophagus aggregatus*), Ri (soil treated with 20 g *R. irregularis*), TN: total nitrogen, TOC: total organic carbon, Ex. = exchangeable. Mean values followed by different letters within the same column are statistically different *(P* < 0.05). The same acronyms apply to other tables and figures.

**Figure 1:**
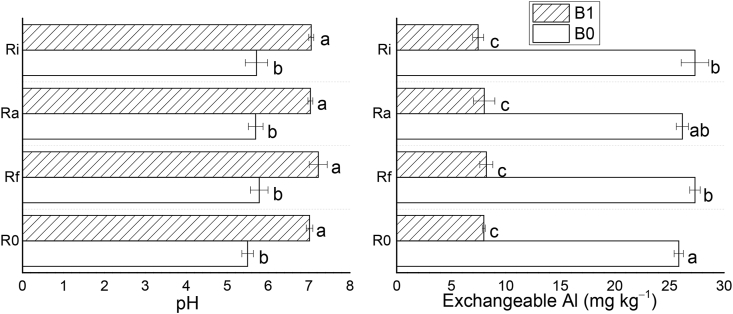
The effect of different amendments on soil exchangeable Al (A) and soil pH (B) during *T. indica* cultivation. Mean values followed by different letters above the bars are statistically different (*P* < 0.05).

After harvesting, the soil pH was reduced by a 1.0 pH unit in the control relative to the original uncultivated soil pH (Table 1 and Figure 1B). This shows that soil cultivation and harvesting of crops also has a negative impact on soil pH as base cations are taken up by the plants. Biochar treatments significantly increased soil pH after cultivation relative to the control while the increase in pH was not significant for AMF treatments (Figure 1B). An important negative consequence of soil acidification is an increase in the concentration of phytotoxic exchangeable Al (Al^3+^). In this study, the content of exchangeable Al^3+^ after the cultivation of *T. indica* in the control treatment was 20.45 mg kg^−1^ (Figure 1A). After treatment with AMF, the content of Al^3+^ was slightly decreased under Rf + B0 and Ra + B0 while it was significantly increased under Ri + B0. This shows that the different fungi interacted differently with Al-bearing minerals in the soil, with Ri promoting the dissolution of these minerals, thus promoting the release of exchangeable Al^3+^. On the other hand, the biochar treatments significantly decreased the content of phytotoxic Al^3+^ by up to >90% and attest to the significant role of biochar in inhibiting soil acidification and retarding Al phytotoxicity [19]. Statistically, biochar and its interaction with AMF significantly correlated with exchangeable Al and pH while AMF did not (Table S1). This suggests that the observed decrease in the content of exchangeable Al may be due to the enhancing effects of different treatments on soil pH.

### 3.2 Impact of biochar amendment with/without AMF on *T. indica* growth parameters

**Figure 2:**
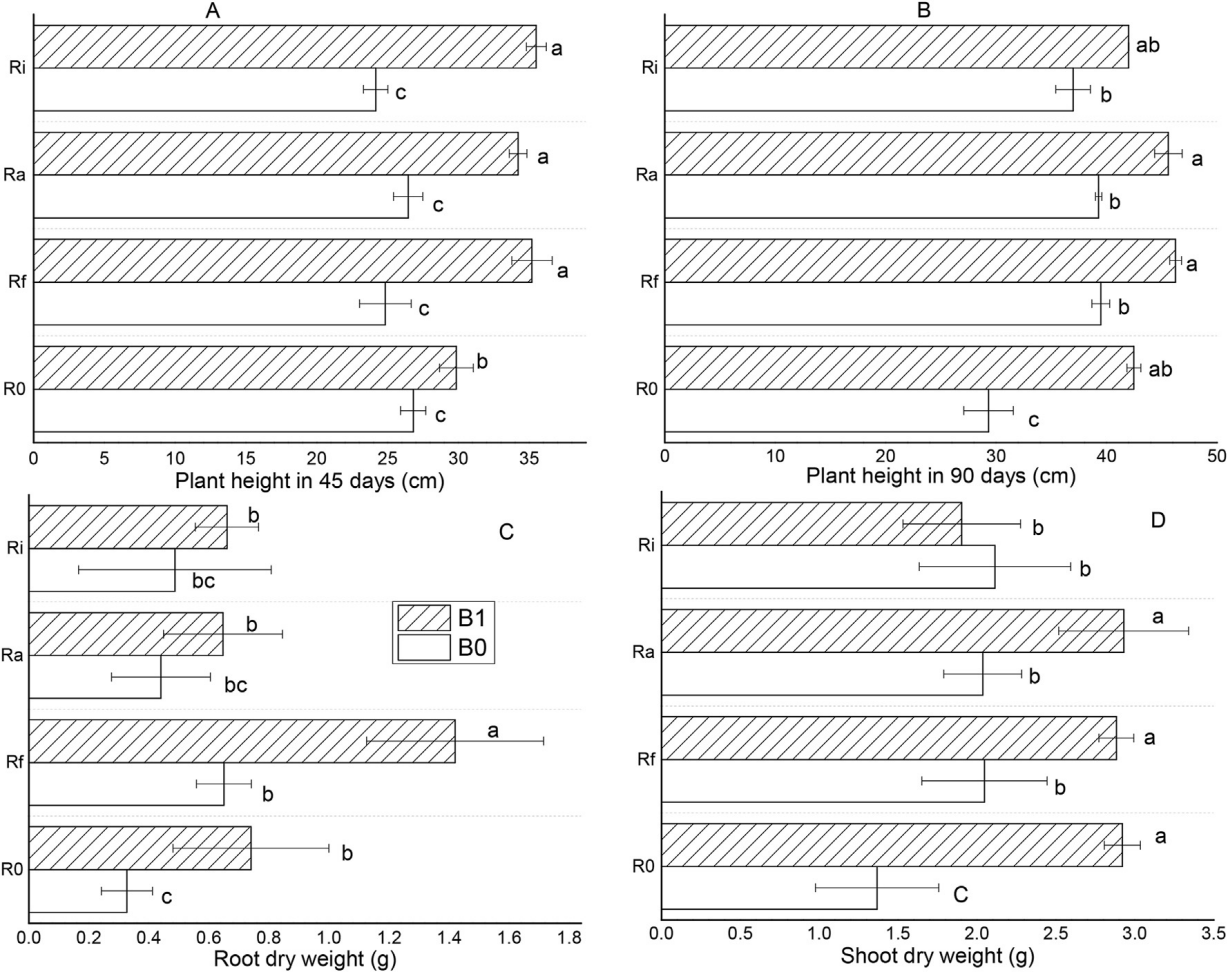
Observed differences in the height after 45 (A) and 90 (B) days of growth and the dry weights of root (C) and shoot (D) of *T. indica* as affected by different amendments and grown in acid and Al-toxic soil. Mean values followed by different letters above the bars are statistically different (*P* < 0.05).

The effects of different amendments on plant growth were evaluated in terms of plant height, shoot and root dry weights after *T. indica* was subjected to Al stress (Figure 2). After 45 days of treatments application (Figure 2A), the plant height was significantly increased under the biochar treatments but not under treatments containing AMF alone relative to the control. After 90 days of growth (Figure 2B), the height of *T. indica* was significantly increased for all the treatments relative to control; with the biochar, treatments showing the most significant effects. Thus, it can be inferred that the slow growth of *T. indica* observed in control and AMF treatments may be related to the phytotoxic effect of Al^3+^ (Figure 1) in these treatments compared to the biochar treatments.

Figure 2 (C and D) shows that there is a significant (*P* < 0.05) difference in the dry weights of the shoot and roots of *T. indica* grown on the amended soils relative to the control. After harvesting on day 90 and drying, it was observed that the shoot and roots of *T. indica* responded differently to acid and Al-toxic soil under the different amendment conditions. The results indicate that the soil amendments played an important role in reducing soil acidity and Al toxicity thereby improving the growth and biomass of *T. indica*. The root dry weight was increased by 126.5, 334.6, 97.9, 102.0, 99.0, 34.7, and 49.0% under B1, B1 + Rf, B1 + Ra, B1 + Ri, Rf + B0, Ra + B0, and Ri + B0 treatments, respectively (Figure 2C). Also, the most significant increase in shoot dry weight was observed under biochar treatments, with a growth rate >100% recorded for B1, B1 + Rf, and B1 + Ra, while B1 + Ri had a 39.3% effect (Figure 2D). For AMF treatments, the percentage increase in shoot dry weight was 49.8, 49.0, and 54.6% for Rf + B0, Ra + B0, and Ri + B0, respectively. Table 3 shows that biochar and its interaction with AMF were significantly correlated with all measured growth parameters while AMF only showed a significant correlation with plant height after 90 days and root dry weight.

**Table 3:** ANOVA on differences in the height after 45 and 90 days of growth and the dry weights of root and shoot of *T. indica* subjected to different amendments and grown in acid and Al-toxic soil.

	Type III Sum of Squares	df	Mean Square	F	Sig.
Height (cm) after 45 days of growth					
Mycorrhization	14.135	3	4.712	3.671	0.035
Biochar	396.013	1	396.013	308.572	0.000
Mycorrhization ∗ Biochar	61.783	3	20.594	16.047	0.000
Error	20.534	16	1.283		
Height (cm) after 90 days of growth					
Mycorrhization	186.682	3	62.227	47.692	0.000
Biochar	366.524	1	366.524	280.907	0.000
Mycorrhization ∗ Biochar	59.438	3	19.813	15.185	0.000
Error	20.877	16	1.305		
Shoot dry weights (g)					
Mycorrhization	1.007	3	0.336	2.870	0.069
Biochar	3.544	1	3.544	30.307	0.000
Mycorrhization ∗ Biochar	2.393	3	0.798	6.820	0.004
Error	1.871	16	0.117		
Root dry weights (g)					
Mycorrhization	1.064	3	0.355	8.084	0.002
Biochar	0.917	1	0.917	20.895	0.000
Mycorrhization ∗ Biochar	0.338	3	0.113	2.571	0.090
Error	0.702	16	0.044		

### 3.3 The content of total nitrogen and total phosphorous in the shoot and roots of *T. indica*

**Figure 3:**
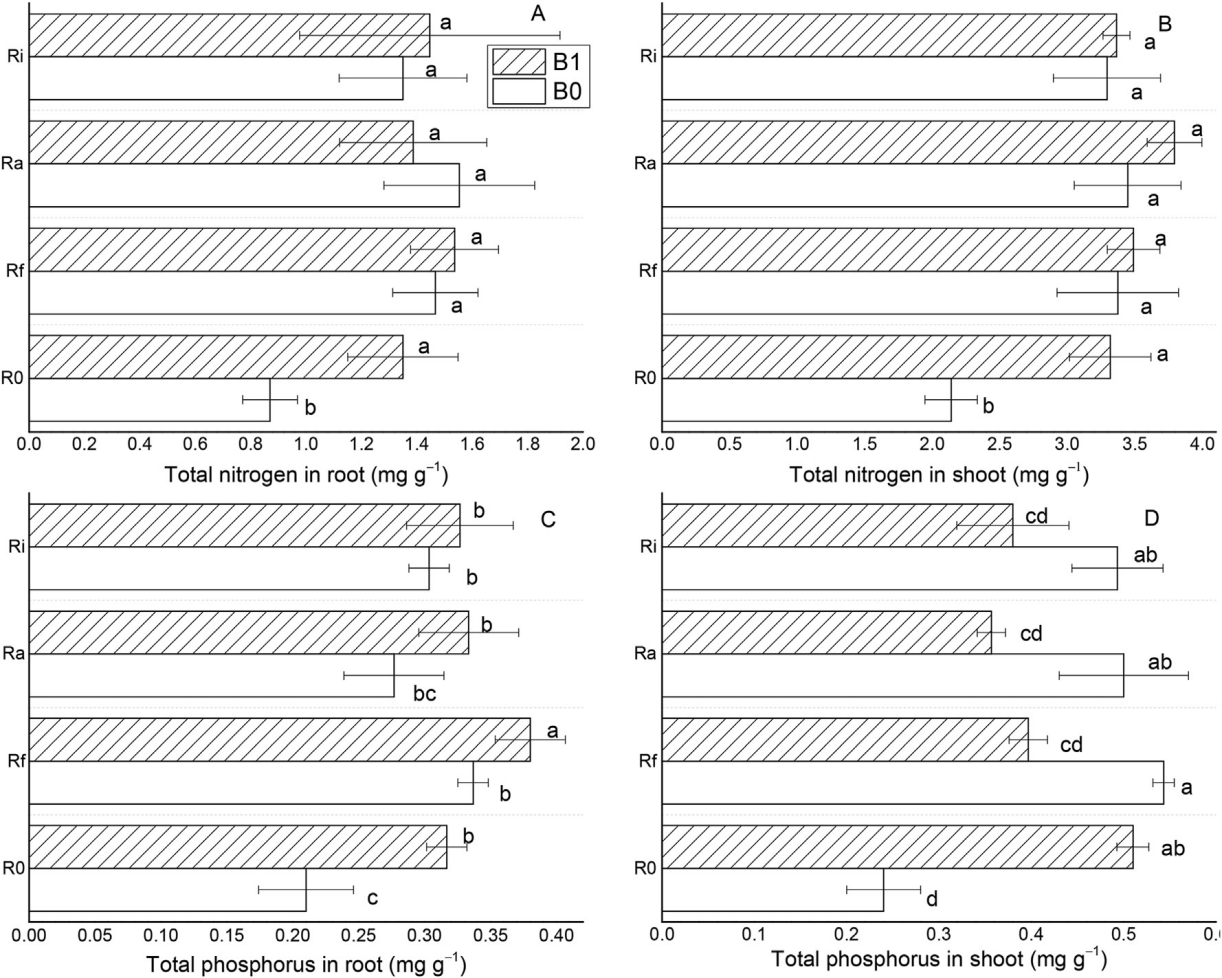
Differences in the contents of total nitrogen in the root (A), shoot (B), total phosphorous in the root (C) and shoot (D) of *T. indica* grown in acid and Al-toxic soil. Mean values followed by different letters above the bars are statistically different (*P* < 0.05).

Figure 3 shows variations in the contents of TN and TP of *T. indica* shoot and roots with the different amendments. It can be observed that the application of biochar and/or AMF highly improved nitrogen (N) and phosphorous (P) contents in the shoot and root tissues of *T. indica* grown in acid and Al-toxic soil. In the shoot, the percent increase in TN and TP was 54.7 and 112.5%, 63.1 and 62.5%, 77.1 and 45.8%, 57.0 and 58.3% for soil amended with B1, B1+ Rf, B1 + Ra, and B1 + Ri, respectively. Comparatively, the corresponding increase in TN and TP when AMF was applied alone was 57.5 and 125%, 60.7 and 108.3%, 53.7 and 104.2% for Rf + B0, Ra + B0, and Ri + B0, respectively (Figures 3C and 3D). Similarly, the percentage increment in TN and TP in the root tissues was 55.2 and 76.2%, 75.9 and 76.2%, 58.6 and 57.1%, 65.5 and 52.4% for amendments with B1, B1 + Rf, B1 + Ra, and B1 + Ri, respectively. Also, for Rf + B0, Ra + B0, and Ri + B0 treatments, the increase in TN and TP was 67.8 and 57.14%, 78.2 and 28.6%, 55.2 and 42.9%, respectively (Figures 3A and 3B). These results show that the application of biochar alone or in combination with AMF does not only alleviate soil acidity and Al toxicity but also improves nutrient uptake by *T. indica*. From Table S2, it can be observed that biochar or AMF treatments showed a significant relationship with TN in plant shoot and TP in plant root while AMF or its interaction with biochar significantly correlated with TP in the shoot.

### 3.4 The impact of different amendments on photosynthetic pigments in the leaves of *T. indica*

**Figure 4:**
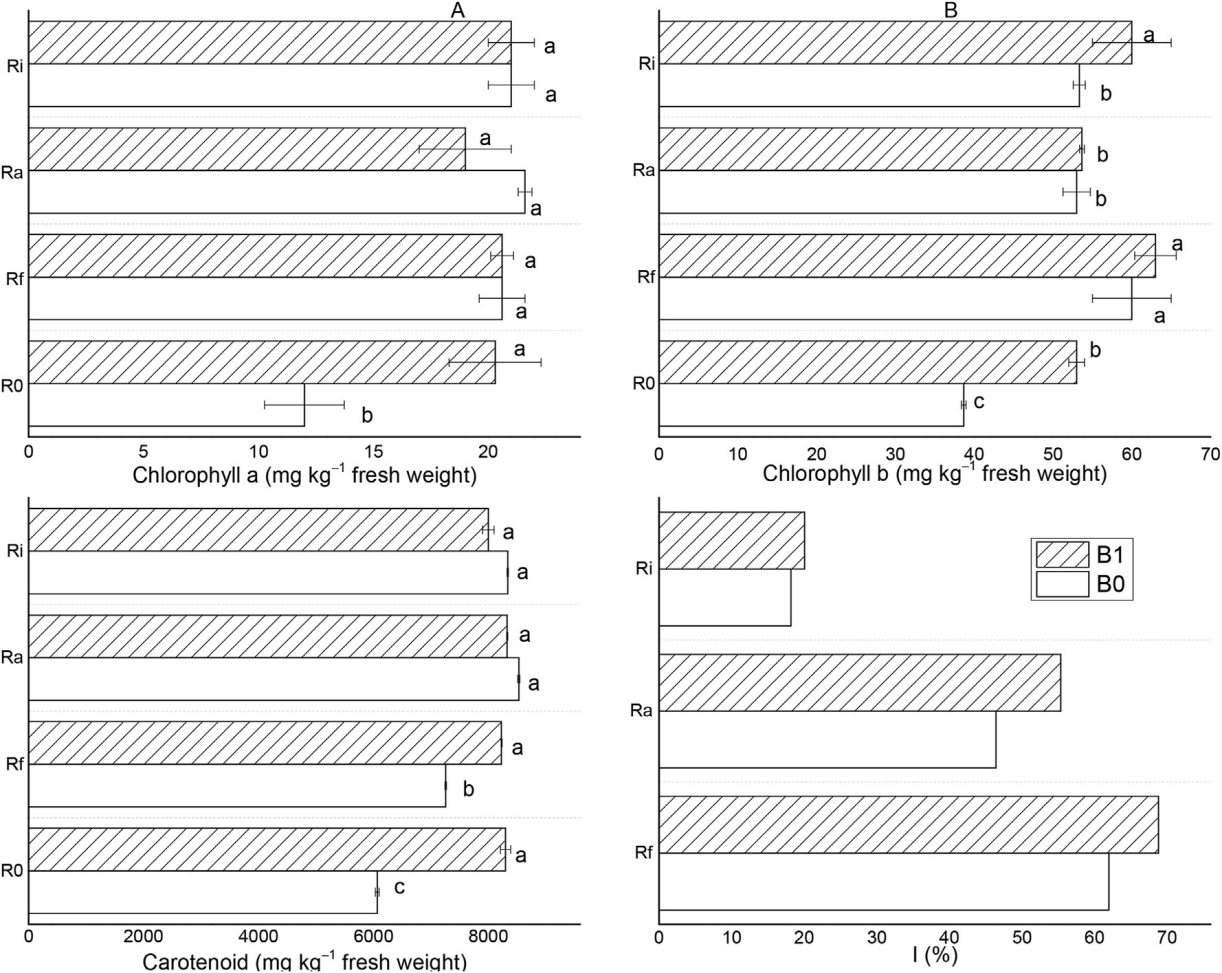
The effects of different amendments on chlorophyll a (A), chlorophyll b (B), carotenoids (C), and the intensity of mycorrhization (D) of *T. indica* grown in acid and Al-toxic soil. Mean values followed by different letters above the bars are statistically different *(P* < 0.05).

Figure 4 shows the content of photosynthetic pigments and how the different amendments affected them. Under conditions of soil acidity and Al toxicity, amending the soil with biochar or biochar + AMF or AMF alone significantly (*P* < 0.05) improved the contents of chlorophyll a/b and carotenoid compared to the control. The percentage increment for chlorophyll a is >50% (Figure 4A), >30% for chlorophyll b (Figure 4B) and >18% for carotenoid (Figure 4C) for all the treatments. Specifically, the contents of chlorophyll a, chlorophyll b, and carotenoid were increased by 69.2%, 35.9%, and 37.0% under biochar treatment, respectively. When the B1 was applied together with Rf, Ra, and Ri, the content of chlorophyll a was increased by 71.7, 58.3, and 75.0% as opposed to 61.5, 35.9, and 53.8% for chlorophyll b and 35.8, 37.5, and 32.0% for carotenoid, respectively. For the AMF treatments alone, the contents of chlorophyll a, chlorophyll b, and carotenoid were increased by 71.7, 53.8, and 19.8% for Rf + B0, 80.0, 35.9, and 40.8% for Ra + B0, 75.0, 35.9, 37.5% for Ri + B0, respectively. This result shows that despite an overall increase in the content of photosynthetic pigments when biochar is applied together with AMF, the magnitude of the increase varies with AMF species; with a combination of B1 + Rf and B1 + Ri having the overall best result. Thus, amending acid soils with biochar or in combination with AMF species can enhance the photosynthetic machinery of *T. indica* under Al stress (Figure 4), and AMF, biochar, or AMF-biochar interactions showed a significant positive correlation with all the photosynthetic pigments (Table S3).

### 3.5 The intensity of mycorrhization and the antioxidant enzymes activity

The intensity of mycorrhization measured in roots of *T. indica* grown in acid and Al-toxic after treatment with AMF and with/without biochar are shown in Figure 4D. Biochar induced an increase in the observed mycorrhization rate relative to the treatments with AMF alone. For instance, the mycorrhization intensities were 62.0, 46.4, and 18.2% for single treatments with Rf, Ra, and Ri, respectively, which increased to 68.8, 55.3, and 20.1% in the presence of biochar, respectively. For all the treatments, B1 + Ra induced the largest increase of 8.9% followed by B1 + Rf (6.8%). These results agree with the increase in plant growth parameters (Figure 2), photosynthetic pigments (Fig. 4A, B, and C), P and N contents in the roots and shoots (Figure 3), soil pH (Figure 1), and TOC (Table 2).

### 3.6 The antioxidant activities of the leaves of *T. indica*

**Figure 5:**
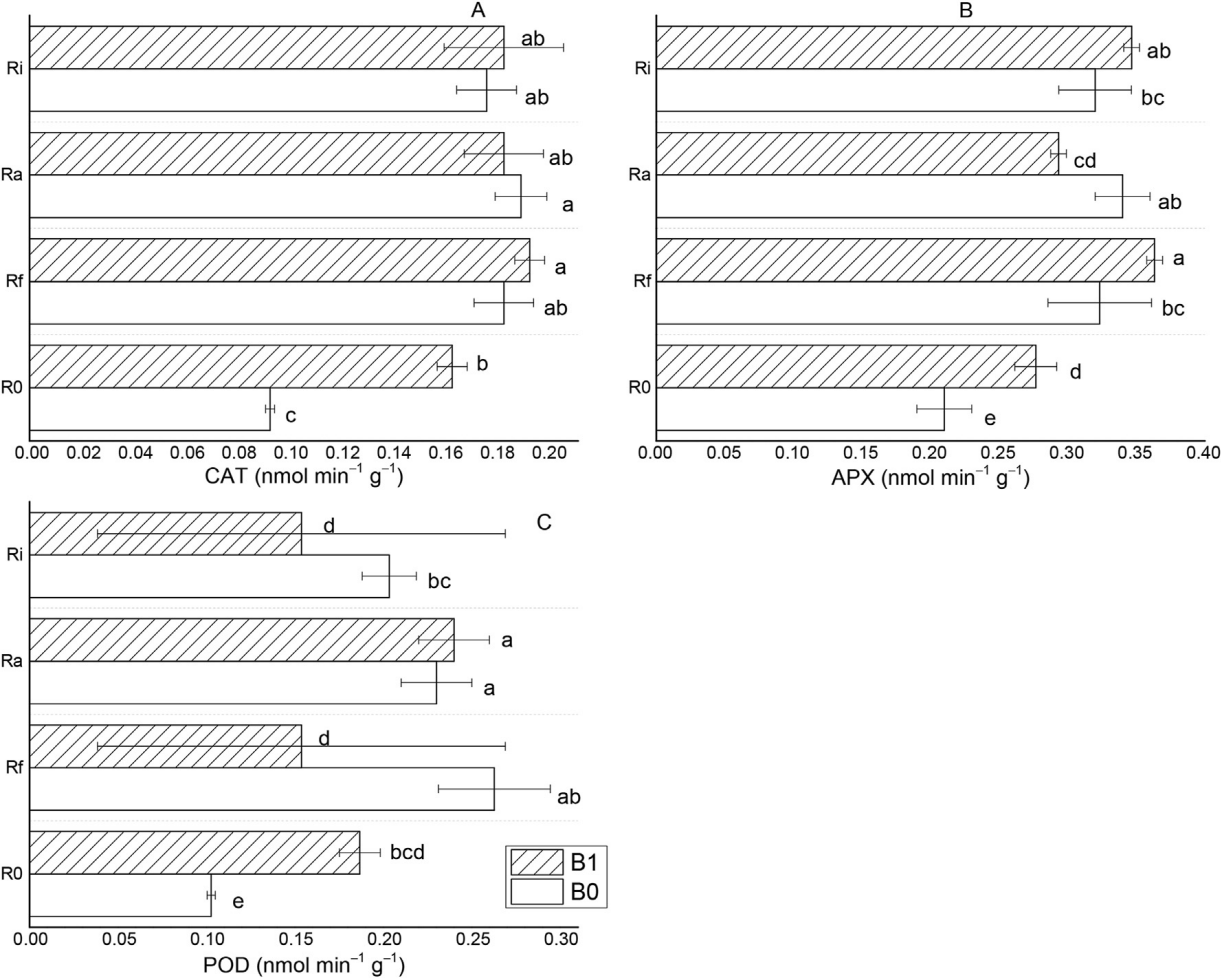
The effects of different amendments on antioxidant activity of the leaves of *T. indica* grown in acid and Al-toxic soil: Catalase (CAT, A), ascorbate peroxidase (APX, B), and guaiacol peroxidase (POD, C). Mean values followed by different letters within the same column are statistically different *(P* < 0.05).

The effect of different amendments on antioxidant activities of the leaves of *T. indica* grown in acid and Al-toxic soil was estimated and the results are shown in Figure 5. The contents of CAT, POD, and SOD were significantly different for the amendments relative to the control. Quantitatively, biochar treatment increased the contents of CAT, POD, and APX by 75.3, 74.8, and 31.4%, respectively. When biochar was applied in combination with AMF, the contents of CAT, POD, and APX were significantly improved; with biochar + Ra having the least increment for APX (39.5%) but the largest for POD (133%). Also, the treatment with B1+ Rf induced the best increase in CAT (107.5%) and APX (72.9%). Generally, the treatments of biochar + AMF showed an average increase in the contents of CAT, POD, and APX by 100.4%, 117.5%, and 59.1%, while treatments with AMF alone had an average of 96.8%, 113.9%, and 56%, respectively. Thus, this result shows that the application of biochar in combination with AMF for the growth of *T. indica* in acidic soils can alleviate the negative impact of Al-induced stress and promote growth. Also, it was observed that the single AMF, biochar, and combined biochar-AMF interactive treatments showed a significant positive correlation with CAT, POD, and APX (Table S4).

## 4 Discussion

In this study, soil acidity negatively affected the growth and functioning of *T. indica* as well as the content of important nutrients required for plant growth. Amending the acidic soil (pH 5.5) with biochar alone or its combination with different AMFs (Rf, Ra, and Ri) induced an increase in the soil pH relative to control treatment. However, after 90 days of growing *T. indica*, the soil pH was decreased by 1.0 pH unit for control, 0.72, 0.81, and 0.78 pH unit for single AMF treatments with Rf + B0, Ra + B0, and Ri + B0, respectively. Under biochar treatments, the final soil pH was increased compared to the original soil pH; 0.51 pH unit for B1, 0.73 pH unit for B1 + Rf, 0.53 pH unit for B1 + Ra, and 0.55 pH unit for B1 + Ri. As a consequence, the content of phytotoxic Al^3+^ was significantly reduced in the biochar treatments thereby inhibiting its adverse effects on root elongation and plant growth parameters. This observation is consistent with the significant positive relationship observed for biochar or biochar-AMF interactions with soil pH and exchangeable Al. The ability of biochar to improve plant growth under adverse conditions have been shown in several studies [63, 64]. In their study, Shi et al. [19] reported that the application of biochar improved root elongation in maize plants under acidification stress by improving soil pH and inhibiting Al^3+^ toxicity. The ability of biochar to improve plant growth is multi-fold as biochar contains important nutrients such as K, Ca, Mg, N, and P which are all essential for plant growth, and when applied to acidic soils, biochar plays the role of retarding the depletion of these nutrients from soils and confirms their significant positive relationship.

The growth parameters of the aboveground *T. indica* recorded after 90 d showed significant differences compared to the control treatment. Within the first 45 d of growth, the single AMF treatments did not improve plant growth compared to the biochar treatments. This was probably because biochar contained the sufficient nutrients required for plant growth while AMF had to provide these nutrients via a series of chemical mechanisms that required more than 45 d to provide enough nutrients. Nevertheless, by the 90^th^ d, the difference in growth rate between biochar treatments and single AMF treatments was not as significant as within the first 45 d. The improvement of soil fertility with biochar is a common agricultural practice nowadays given its high nutrient content [65]. The use of biochar to mitigate the adverse effects of cadmium (Cd) pollution on the growth of tomato plants (*Solanum lycopersicum* L.) revealed that biochar significantly improved the shoot and root dry matter [66]. Similar observations were reported by Ren et al. [67] who studied the mitigating effect of biochar on tobacco grown under Cd-stress. Our results show that biochar treatments and single AMF treatments improved the dry matter weights of the shoot and root of *T*. *indica* and improved nutrient uptake under conditions of soil acidity and Al toxicity stress. This could mean that the different treatments did not only improve the soil nutrient content but facilitated nutrient uptake by *T. indica* under stress-related conditions. In general, the average increase in shoot and root dry weights was 94.6% and 165.3% for biochar treatments as opposed to 51.1% and 60.9% for single AMF treatments. After 45 days, only the biochar and biochar-AMF treatments showed a significant correlation with plant height while after 90 days, all treatments had significant positive correlations with plant height, shoot and root dry weights.

Growth retardation in the control treatment may be attributed to the cumulative influence of both soil acidity stress and Al toxicity, resulting in stunted roots and inhibition of roots cell division/elongation [68, 69]. This is evident in the deficiency of vital nutrients for efficient uptake and subsequently limits nutrient use efficiency and retards photosynthesis in *T. indica*. In their respective studies with *C. arietinum* and maize, Mohammad et al. [70] and Mau and Utami [71] observed that nutrient use deficiency has a direct impact on the plant photosynthesis rate and subsequent tolerance to stress. Also, Gajewska et al. [72] reported that Al-mediated peroxidation of chloroplast membranes can result in chlorophyll degradation and photosynthesis inhibition. Our results indicate lower contents of carotenoids and chlorophyll (a and b) in the control treatment relative to biochar treatments or single AMF treatments. As evident, the larger content of toxic Al^3+^ in the control induced a negative impact on the photosynthesis rate compared to other treatments. Even with similar content of Al^3+^ like the control, the single AMF treatments improved photosynthesis. This suggests that the extracted Al was probably made up of less toxic free Al^3+^ and more complexed species (Al-organic matter and Al-hydroxide) that were less toxic to *T. indica* [73] or AMF enhanced the activity of transport proteins in cell membranes to improve cellular division and cell wall expansion [74, 75]. For all single and interactive treatments, a significant positive relationship exists with all the measured photosynthetic parameters, confirming the ability of biochar, AMF, and biochar-AMF to alleviate Al^3+^ stress and improve soil nutrient content was important for plant growth. Wang et al. [65] reported that the mycorrhization of *Quercus mongolica* seedlings by *Tuber melanosporum* significantly altered the root carbon exudation potential. The authors also observed a 69%, 94%, 0.4 pH unit, 76% improvement in the leaf photosynthetic rate, P concentration, soil pH, and TOC, respectively. In another study, Nahberger et al. [76] showed that earthworms improved mycorrhization of silver fir with *Tuber aestivum*, and the effect was only significant after six months. According to them, the effect became insignificantly negative by the 12 months due to grazing of the root tips. In this study, the least mycorrhization intensity (I%) was observed for Ri amendments. The addition of biochar along with AMF increased the I% by 6.53%, 8.93%, and 1.89% for Rf, Ra, and Ri, respectively. The increase in I% in the biochar treatments corresponds to a larger increase in soil pH, TOC, P-OLSEN, TN, and NO_3_^-^-N/NH_4_^+^-N, and agrees with the results of Wang et al. [65].

Plants have different mechanisms to alleviate stress from different sources. Under heavy metals (HMs)-induced stress, for example, plants can initiate different defense mechanisms by producing POD and CAT for example [67]. In this study, soil acidity and Al toxicity-related stresses were observed in the control treatment as the growth parameters were significantly lower compared to other treatments. Our data reveal that the different soil amendments significantly increased the activities of the antioxidant enzymes (CAT, POD, and APX) relative to our control treatment. This result suggests that the different amendments can mitigate drought-related stress by reducing the oxidative damage caused by soil acidity and/or Al toxicity by influencing the plant antioxidant system [49]. Numerous studies have shown that antioxidants CAT, POD, and APX are indispensable enzymes for the cellular defense mechanism against excessive reactive oxygen species (ROS) production in cells [77]. These antioxidants play a key role in scavenging ROS concentrations and in alleviating oxidative stress in plant cells [78]. Latef et al. [79] suggested that improving antioxidant enzymes activities of mycorrhizal plants enhanced plant growth by mitigating oxidative stress. Hence, the proposed treatments in this study can play a key role in alleviating growth-inhibiting stress and promoting the growth of *T. indica* in acidic soils. This is confirmed by the significant positive relationship observed between the different single and interactive treatments with the contents of CAT, APX, and POD.

Few reports have analyzed the combined treatments of AMF and biochar in alleviating the detrimental impact of acid and Al-toxic soils in forest plants in semi-arid regions [55]. It was revealed that *Catalpa bungei* roots colonization decreased when soils were amended exclusively with AMF [80]. The intensity of colonization evaluated in amended soils in this study shows the beneficial impact of biochar on AMF colonization and host plant protection and agrees with Yusif et al. [81] who suggested that amending soils with biochar improves the physicochemical characteristics of soils and make them more conducive for AMF colonization. Biochar derived from the wood of *Eucalyptus deglupta* improved the growth of common beans (*Phaseolus vulgaris*) by improving soil water holding capacity and cation exchange capacity [82]. Wu et al. [83] reported that AMF can mitigate the adverse effects of abiotic stress by reducing oxidative stress and thereby improve the growth of the citrus plant. Soil moisture content plays a key role during mycorrhizal colonization given that AMF spores in soils are required to germinate and grow [84]. Therefore, co-amendments such as biochar with the potential to improve soil moisture content will enhance the mycorrhization efficiency and improve plant growth.

While we worry about drought-related stress to agriculture in arid and semi-arid regions of the world, other factors such as soil acidity and Al toxicity occur to intensify the effects of drought stress on forest species and have a significant negative impact on the physiological and biochemical foundations of plant metabolism [8, 85]. Biochar amendments [86, 87] and AMF inoculation [70, 88, 89] have been reported to generate various beneficial effects on plants ranging from morphology to metabolism, as a result of their involvement in modifying the soil environment [90, 91]. As evident in this study, amending an acidic soil with biochar alone, biochar + AMF, and AMF alone demonstrated a positive effect in modifying the soil environment and improving the growth of *T. indica*.

## 5 Conclusion

Amending an acidic soil with biochar and/or AMF improved soil nutrient content, ameliorated soil acidity, enhanced nutrient uptake, and increased the above and below-ground biomass of *T. indica* cultivated under acid and Al-toxic soil. The overall effect of the amendments was significant for treatments containing biochar. Also, the application of biochar with AMF improved the colonization potential of AMF and significantly increased the photosynthetic potential of *T. indica* by enhancing the contents of chlorophyll and carotenoids. In addition, the different amendments mitigated stress-induced oxidative stress by improving the activities of antioxidant enzymes CAT, POD, and APX. Nevertheless, more long-term studies are needed for different plant species in both greenhouse and field conditions to conclude whether biochar and/or AMF application can effectively mitigate the adverse effects of soil acidity and Aluminum toxicity-related stress on plant growth.

## Declarations

### Author contribution statement

Ndiaye Ibra Ndiate: Conceived and designed the experiments; Performed the experiments; Analyzed and interpreted the data; Wrote the paper.

Cai Li Qun; Jackson Nkoh Nkoh: Contributed reagents, materials, analysis tools or data.

### Funding statement

This work was supported by 10.13039/501100001809National Natural Science Foundation of China (41661049, 31571594), and the Scientific Research Start-up Funds for Openly-Recruited Doctors.

### Data availability statement

Data will be made available on request.

### Declaration of interests statement

The authors declare no conflict of interest.

### Additional information

No additional information is available for this paper.
