# Supplementary material for: Importance of soil amendments with biochar and/or Arbuscular Mycorrhizal fungi to mitigate aluminum toxicity in tamarind (Tamarindus indica L.) on an acidic soil: A greenhouse study
Source: Heliyon. 2022 Feb 23;8(2):e09009. doi: 10.1016/j.heliyon.2022.e09009 (PMC8886007; doi:10.1016/j.heliyon.2022.e09009)
Supplement: Supplementary material 1 [file mmc1.doc]

**Table S1.** ANOVA on the effect of soil amendments on soil chemical characteristics after harvesting of *T. indica*.

| TN (g kg^-1^) | | | | | |
| --- | --- | --- | --- | --- | --- |
| Type III Sum  of Squares df Mean Square F Sig. | | | | | |
| Mycorrhization | 0.017 | 3 | 0.006 | 0.262 | 0.852 |
| Biochar | 0.288 | 1 | 0.288 | 13.108 | 0.002 |
| Mycorrhization * Biochar | 1.284 | 3 | 0.428 | 19.468 | 0.000 |
| Error | 0.352 | 16 | 0.022 |  |  |
| NH_4_-N (mg kg^-1^) | | | | | |
| Type III Sum  of Squares df Mean Square F Sig. | | | | | |
| Mycorrhization | 0.773 | 3 | 0.258 | 0.157 | 0.924 |
| Biochar | 152.949 | 1 | 152.949 | 93.328 | 0.000 |
| Mycorrhization * Biochar | 496.520 | 3 | 165.507 | 100.990 | 0.000 |
| Error | 26.221 | 16 | 1.639 |  |  |
| NO_3_-N (mg kg^-1^) | | | | | |
| Type III Sum  of Squares df Mean Square F Sig. | | | | | |
| Mycorrhization | 0.765 | 3 | 0.255 | 0.334 | 0.801 |
| Biochar | 566.171 | 1 | 566.171 | 740.775 | 0.000 |
| Mycorrhization * Biochar | 2094.441 | 3 | 698.147 | 913.452 | 0 .000 |
| Error | 12.229 | 16 | 0.764 |  |  |
| TOC (%) | | | | | |
| Type III Sum  of Squares df Mean Square F Sig. | | | | | |
| Mycorrhization | 0.028 | 3 | 0.009 | 0.300 | 0.825 |
| Biochar | 0.010 | 1 | 0.010 | 0.331 | 0.573 |
| Mycorrhization * Biochar | 0.564 | 3 | 0.188 | 5.953 | 0.006 |
| Error | 0.505 | 16 | 0.032 |  |  |
| P-OLSEN (g kg^-1^)  Type III Sum  of Squares df Mean Square F Sig. | | | | | |
| Mycorrhization | 1.180 | 3 | 0.393 | 0.305 | 0.822 |
| Biochar | 3.042 | 1 | 3.042 | 2.356 | 0.144 |
| Mycorrhization * Biochar | 10.489 | 3 | 3.496 | 2.708 | 0.080 |
| Error | 20.657 | 16 | 1.291 |  |  |
| Ex. Ca (g kg^-1^) | | | | | |
| Type III Sum  of Squares df Mean Square F Sig. | | | | | |
| Mycorrhization | 0.339 | 3 | 0.113 | 0.174 | 0.912 |
| Biochar | 0.844 | 1 | 0.844 | 1.300 | 0.271 |
| Mycorrhization * Biochar | 0.774 | 3 | 0.258 | 0.397 | 0.757 |
| Error | 10.387 | 16 | 0.649 |  |  |
| Ex. Mg (g kg^-1^) | | | | | |
| Type III Sum  of Squares df Mean Square F Sig. | | | | | |
| Mycorrhization | 0.527 | 3 | 0.176 | 0.727 | 0.551 |
| Biochar | 1.245 | 1 | 1.245 | 5.150 | 0.037 |
| Mycorrhization * Biochar | 0.089 | 3 | 0.030 | 0.123 | 0.945 |
| Error | 3.868 | 16 | 0.242 |  |  |
| Ex. K (g kg^-1^) | | | | | |
| Type III Sum  of Squares df Mean Square F Sig. | | | | | |
| Mycorrhization | 0.024 | 3 | 0.008 | 0.551 | 0.655 |
| Biochar | 0.087 | 1 | 0.087 | 5.981 | 0.026 |
| Mycorrhization * Biochar | 0.009 | 3 | 0.003 | 0.197 | 0.897 |
| Error | 0.231 | 16 | 0.014 |  |  |
| Ex. Al (mg kg^-1^) | | | | | |
| Type III Sum  of Squares df Mean Square F Sig. | | | | | |
| Mycorrhization | 4.146 | 3 | 1.382 | 2.847 | 0.070 |
| Biochar | 548.495 | 1 | 548.495 | 1129.747 | 0.000 |
| Mycorrhization * Biochar | 1564.029 | 3 | 521.343 | 1073.820 | 0.000 |
| Error | 7.768 | 16 | 0.486 |  |  |
| pH | | | | | |
| Type III Sum  of Squares df Mean Square F Sig. | | | | | |
| Mycorrhization | 0.105 | 3 | 0.035 | 1.199 | 0.342 |
| Biochar | 2.385 | 1 | 2.385 | 81.567 | 0.000 |
| Mycorrhization * Biochar | 9.624 | 3 | 3.208 | 109.729 | 0.000 |
| Error | 0.468 | 16 | 0.029 |  |  |

**Table S2.** ANOVA on differences in the contents of total nitrogen (TN) and total phosphorous (TP) in the shoot and root of *T. indica* grown in acid and Al-toxic soil.

| TN in the shoot (mg g-1) | | | | | |
| --- | --- | --- | --- | --- | --- |
| Type III Sum  of Squares df Mean Square F Sig. | | | | | |
| Mycorrhization | 2.671 | 3 | 0.890 | 9.704 | 0.001 |
| Biochar | 1.097 | 1 | 1.097 | 11.950 | 0.003 |
| Mycorrhization * Biochar | 1.188 | 3 | 0.396 | 4.317 | 0.021 |
| Error | 1.468 | 16 | 0.092 |  |  |
| TP in the shoot (mg g-1) | | | | | |
| Type III Sum  of Squares df Mean Square F Sig. | | | | | |
| Mycorrhization | 0.028 | 3 | 0.009 | 5.414 | 0.009 |
| Biochar | 0.007 | 1 | 0.007 | 3.883 | 0.066 |
| Mycorrhization * Biochar | 0.185 | 3 | 0.062 | 35.929 | 0.000 |
| Error | 0.027 | 16 | 0.002 |  |  |
| TN in the root (mg g-1) | | | | | |
| Type III Sum  of Squares df Mean Square F Sig. | | | | | |
| Mycorrhization | 0.574 | 3 | 0.191 | 2.960 | 0.064 |
| Biochar | 0.086 | 1 | 0.086 | 1.336 | 0.265 |
| Mycorrhization * Biochar | 0.322 | 3 | 0.107 | 1.661 | 0.215 |
| Error | 1.035 | 16 | 0.065 |  |  |
| TP in the roots (mg g-1) | | | | | |
| Type III Sum  of Squares df Mean Square F Sig. | | | | | |
| Mycorrhization | 0.027 | 3 | 0.009 | 10.283 | 0.001 |
| Biochar | 0.020 | 1 | 0.020 | 22.352 | 0.000 |
| Mycorrhization * Biochar | 0.006 | 3 | 0.002 | 2.133 | 0.136 |
| Error | 0.014 | 16 | 0.001 |  |  |

**Table S3**. ANOVA on the effects of different amendments on chlorophyll a, chlorophyll b, carotenoids, and the intensity of mycorrhization of *T. indica* grown in acid and Al-toxic soil.

| Chlorophyll a (mg g-1 fresh weight) | | | | | |
| --- | --- | --- | --- | --- | --- |
| Type III Sum  of Squares df Mean Square F Sig. | | | | | |
| Mycorrhization | 9.193E-05 | 3 | 3.064E-05 | 17.096 | 0.000 |
| Biochar | 1.218E-05 | 1 | 1.218E-05 | 6.797 | 0.019 |
| Mycorrhization * Biochar | 0.000 | 3 | 3.376E-05 | 18.836 | 0.000 |
| Error | 2.868E-05 | 16 | 1.793E-06 |  |  |
| Chlorophyll b (mg g-1 fresh weight) | | | | | |
| Type III Sum  of Squares df Mean Square F Sig. | | | | | |
| Mycorrhization | 0.001 | 3 | 0.000 | 33.699 | 0.000 |
| Biochar | 0.000 | 1 | 0.000 | 29.560 | 0.000 |
| Mycorrhization * Biochar | 0.000 | 3 | 5.361E-05 | 6.946 | 0.003 |
| Error | 0.000 | 16 | 7.719E-06 |  |  |
| Carotenoids (mg g-1 fresh weight) | | | | | |
| Type III Sum  of Squares df Mean Square F Sig. | | | | | |
| Mycorrhization | 5.341 | 3 | 1.780 | 714.503 | 0.000 |
| Biochar | 2.660 | 1 | 2.660 | 1067.560 | 0.000 |
| Mycorrhization * Biochar | 6.466 | 3 | 2.155 | 865.072 | 0.000 |
| Error | 0.040 | 16 | 0.002 |  |  |

**Table S4**. ANOVA on the effects of different amendments on antioxidant activity of the leaves of *T. indica* grown in acid and Al-toxic soil: Catalase (CAT), ascorbate peroxidase (APX), and guaiacol peroxidase (POD).

| CAT (nmol min-1 g-1) | | | | | |
| --- | --- | --- | --- | --- | --- |
| Type III Sum  of Squares df Mean Square F Sig. | | | | | |
| Mycorrhization | 0.015 | 3 | 0.005 | 32.737 | 0.000 |
| Biochar | 0.002 | 1 | 0.002 | 16.093 | 0.001 |
| Mycorrhization * Biochar | 0.005 | 3 | 0.002 | 11.712 | 0.000 |
| Error | 0.002 | 16 | 0.000 |  |  |
| POD (nmol min-1 g-1) | | | | | |
| Type III Sum  of Squares df Mean Square F Sig. | | | | | |
| Mycorrhization | 0.027 | 3 | 0.009 | 2.520 | 0.095 |
| Biochar | 0.002 | 1 | 0.002 | 0.436 | 0.518 |
| Mycorrhization * Biochar | 0.031 | 3 | 0.010 | 2.844 | 0.071 |
| Error | 0.058 | 16 | 0.004 |  |  |
| APX (nmol min-1 g-1) | | | | | |
| Type III Sum  of Squares df Mean Square F Sig. | | | | | |
| Mycorrhization | 0.037 | 3 | 0.012 | 30.082 | 0.000 |
| Biochar | 0.003 | 1 | 0.003 | 6.898 | 0.018 |
| Mycorrhization * Biochar | 0.011 | 3 | 0.004 | 8.639 | 0.001 |
| Error | 0.007 | 16 | 0.000 |  |  |
